# Rescue medication use as a patient-reported outcome in COPD: a systematic review and regression analysis

**DOI:** 10.1186/s12931-017-0566-1

**Published:** 2017-05-08

**Authors:** Yogesh Suresh Punekar, Sheetal Sharma, Ankit Pahwa, Jitender Takyar, Ian Naya, Paul W. Jones

**Affiliations:** 1 Health Outcomes, ViiV Healthcare, 980 Great West Road, Brentford, Middlesex, TW8 9GS UK; 2 PAREXEL® Access Consulting, PAREXEL® International, Chandigarh, India; 3 Respiratory Medical, GSK, Brentford, Middlesex, UK; 4 Institute of Infection and Immunity, St George’s, University of London, London, UK

**Keywords:** COPD, Long-acting β_2_-agonist, Long-acting muscarinic antagonist, Inhaled corticosteroid, Lung function outcomes, Rescue medication use, Patient-reported outcomes

## Abstract

**Background:**

Reducing rescue medication use is a guideline-defined goal of asthma treatment, however, little is known about the validity of rescue medicine use as a marker of symptoms in chronic obstructive pulmonary disease (COPD). To improve patient outcomes, greater insight is needed into the relationship between rescue medication use and alternative COPD outcomes.

**Methods:**

A systematic search of electronic databases (Embase®, MEDLINE® and Cochrane CENTRAL) was conducted from database start to 26 May, 2015. Studies of bronchodilator therapy with a duration of ≥24 weeks were included if they reported either mean change from baseline (CFB) in rescue medication use in puffs/day or % rescue-free days (%RFD), and at least one other COPD endpoint. Correlation and meta-regression analyses were undertaken to test the association between rescue medication use and other COPD outcomes using weighted means (weights proportional to the sample size of the treatment group) and unweighted means (equal weight for each treatment group). Each association was assessed at 6 months and study end.

**Results:**

Forty-six studies involving 46,531 patients provided mean data from 145 treatment groups for evaluation. Changes in both measures of rescue medication use were correlated with changes in trough forced expiratory volume in one second ([FEV_1_]; Pearson correlation coefficients |r| ≥ 0.63; *p* < 0.0001) and with St George’s Respiratory Questionnaire (SGRQ) score (|r| ≥ 0.70; *p* < 0.0001) at study end. Change in rescue medication use in puffs/day during the study correlated with annualized rates of moderate/severe exacerbations at 6 months and study end (both *r* = 0.66; *p* ≤ 0.0028). CFB in puffs/day was not well correlated with Transition Dyspnoea Index (TDI), but %RFD did correlate with TDI score at 6 months and study end (both *r* = 0.69; *p* < 0.0001). The values for CFB in puffs/day corresponding to the proposed minimal clinically important differences for trough FEV_1_ and SGRQ score were -1.3 and -0.6 puffs/day, respectively. A -1.0 puffs/day CFB in rescue use corresponded to a change of 0.26 events/patient-year in moderate/severe exacerbations.

**Conclusion:**

This analysis provides clear evidence of associations at a patient group level between rescue medication use and other clinically important COPD outcomes.

**Electronic supplementary material:**

The online version of this article (doi:10.1186/s12931-017-0566-1) contains supplementary material, which is available to authorized users.

## Background

Chronic obstructive pulmonary disease (COPD) is a progressive disease of the respiratory system and is characterized by chronic airway inflammation [1, 2]. Spirometry assessments are important for the characterization and management of COPD, but the Global initiative for chronic Obstructive Lung Disease (GOLD) guidelines recommend that the symptoms experienced by each individual patient and their exacerbation history should also be considered [2]. Furthermore, individual studies and meta-analyses have shown variable levels of correlation between the spirometric measure of forced expiratory volume in one second (FEV_1_) and health status scores [3–6]. For example, in the TORCH study, a deterioration in health status (assessed by the St George’s Respiratory Questionnaire [SGRQ]) at 3 years correlated significantly but relatively poorly with change in FEV_1_ (*r* = −0.24; *p* < 0.0001) [4]. A more recent meta-analysis that assessed spirometric measurements and patient-reported outcomes found a much stronger correlation between trough FEV_1_ and SGRQ (*r* = -0.68; *p* < 0.0001) [6].

Rescue medication use is commonly measured in clinical trials of patients with asthma and COPD. However, whilst asthma guidelines focus on minimizing the need for rescue therapy [7], far less emphasis is placed on rescue therapy in COPD. To the best of our knowledge, no previous systematic reviews have assessed the association between increased rescue medication use and other clinical trial outcomes such as changes in trough FEV_1_, incidence of moderate-to-severe COPD exacerbations or patient-reported outcomes such as SGRQ or the Transition Dyspnoea Index (TDI) [5, 6]. Recently, increased levels of rescue medication use and temporal changes in its use have been linked with increased exacerbation risk in patients with moderate-to-very-severe COPD [3]. Whether this finding can be confirmed across multiple randomized controlled trials (RCTs) remains to be established. The primary objective of the current analysis was to assess the relationship between rescue medication use and other COPD outcomes measured in clinical trials.

## Methods

### Sources

**Fig. 1:**
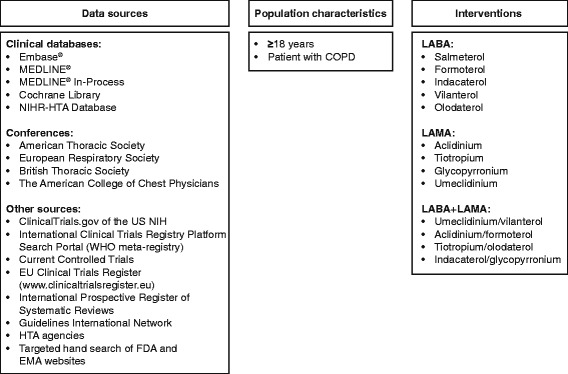
Data sources and selection criteria for the review. *Abbreviations*: *COPD* chronic obstructive pulmonary disease; *EMA* European Medicines Agency; *Embase* Excerpta Medica Database; *FDA* Food and Drug Administration; *HTA* Health Technology Assessment; *ICS* inhaled corticosteroid; *LABA* long-acting β_2_-agonist; *LAMA* long-acting muscarinic antagonist; *MEDLINE* Medical Literature Analysis and Retrieval System Online; *NIHR* National Institute for Health Research; *WHO* World Health Organization

Systematic searches of the literature were performed using a predefined search strategy to identify studies in COPD. A range of data sources were searched from database start to 26 May, 2015, including clinical trial databases, clinical trial registries and conferences, and the results were supplemented with information retrieved from other relevant websites (Fig. 1). This review included a new analysis of data from a previous systematic literature review [6] and data found in a subsequent systematic literature search.

### Search strategy and selection criteria

Specific search strings were devised for the databases to be searched and are shown in Additional file 1: Table S1. They broadly encompassed the terms ‘COPD’ and the interventions for inclusion (long-acting β_2_-agonists [LABAs]: formoterol, indacaterol, olodaterol, salmeterol, vilanterol; long-acting muscarinic antagonists [LAMAs]: aclidinium, glycopyrronium, tiotropium, umeclidinium; LABA + LAMA: aclidinium/formoterol, indacaterol/glycopyrronium, tiotropium/olodaterol, umeclidinium/vilanterol).

The inclusion criteria employed in this analysis were consistent with those employed in the previous analysis [6]. Only studies with the full texts published in English language were included. Eligible studies were RCTs of ≥24 weeks’ duration that enrolled patients of any gender or race, who were aged ≥18 years and had COPD (as defined by GOLD criteria [2]). Studies that enrolled a mixed population of patients with asthma and COPD were only included if subgroup data were available for the COPD population. Case series, case studies and case reports were excluded due to their smaller size, non-comparative nature and higher risk of bias versus RCTs. Only studies that reported rescue medication use and at least one other outcome used in COPD trials were included. The rescue medication data included were expressed as change from baseline in either mean puffs/day or percentage of rescue-free days. Other outcomes used in COPD included were: trough FEV_1_, TDI focal score, SGRQ total score and annualized exacerbation rate. Eligible studies included at least one treatment arm with an inhaled LABA, LAMA or LABA + LAMA combination. Studies that included a treatment of interest in combination with treatments not of interest, such as an inhaled corticosteroid (ICS)/LABA combination, without an additional LAMA, LABA or LAMA + LABA arm, were excluded. There were no other restrictions on the inclusion of studies.

Bibliographic details, abstracts and titles of all citations identified by the literature search were imported into a database, which was used for first pass and second pass of the citations, as well as data extraction. Two independent reviewers first screened the abstracts of all citations (first pass); if they met the study selection criteria, the full-text citations were screened (second pass). Any discrepancies between the reviewers’ decisions at either stage were resolved by a third independent reviewer. Any duplicates of citations were also excluded at first pass stage; any similar publications containing the same data were linked at the publication stage. Risk of bias in studies was assessed at the study level using a critical appraisal checklist [8].

### Data extraction

Data extraction was performed using a standardized pre-piloted data extraction form. Data were extracted in parallel by two independent reviewers from the text and tables of documents for the following time points: baseline, 6 months and study end. In cases where data were only available in figures, data were extracted using Engauge Digitiser software. For each study treatment group, endpoint of interest, and selected time points, mean change from baseline, mean baseline and mean follow-up values were extracted. In cases where mean changes from baseline values were not available, these were calculated at the analysis stage from the mean value at follow-up minus the mean value at baseline. A review of the distribution of all values was conducted by the researchers and analytical lead to identify outliers, which were cross-checked against the source document and confirmed or modified if required.

### Statistical analysis

Study and patient characteristics, as well as outcome results, were described at treatment group level and summarized at study level across all studies. These data were summarized within and across studies using equal weights for each study treatment group (unweighted approach) and by using weights proportional to the study treatment group size. Weights were calculated as the treatment group sample size divided by the total number of patients across all treatment groups. The primary analysis and data interpretation used the weighted data.

Pearson correlation and linear regression analyses were used to assess the relationship between rescue medication use and COPD outcomes and were performed separately for all trials. Analyses were conducted only for the combination of outcomes for which data from at least 15 study treatment groups were available. The sample size allowed the detection of a correlation coefficient of 0.7 with 90% power and an associated type I error of 0.05.

Pearson correlation coefficients (weighted analysis) were determined with their 95% confidence intervals (CI). Correlation coefficients were weighted by study treatment group sample size to compensate for variability in sample sizes across studies. Interpretation of the amplitude of the absolute values of correlation coefficients were based on Cohen’s conventions (0.1–0.3, small/weak; 0.3–0.5, medium/moderate; >0.5, large) [8, 9]. The associations between rescue medication use and COPD outcomes were represented visually using bubble plots (weighted analysis). Each bubble represents a study treatment group and the size of a bubble is proportional to the study treatment group sample size divided by the total number of patients across all studies.

Linear regression analysis was conducted to identify the strength and direction of association between rescue medication use and COPD outcomes and to estimate rescue medication use values corresponding to proposed minimal clinically important difference (MCID) values for COPD outcomes. Regression coefficients were determined using least squares means and the coefficient of determination (*R* ^2^) was calculated.

All data processing and analyses were performed with SAS software v11.2 (SAS Institute, Cary, NC, USA).

## Results

### Overview and characteristics of included studies

**Fig. 2:**
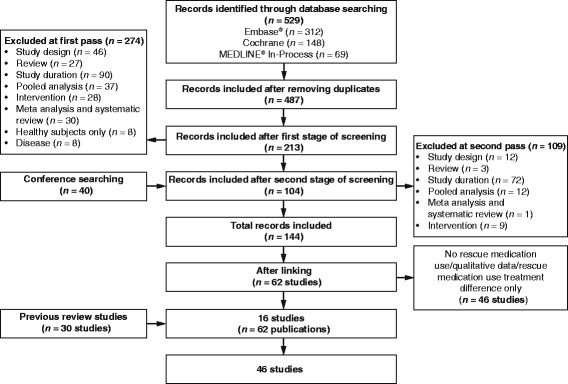
Results of the literature search and evaluation of identified studies according to PRISMA. *Abbreviations*: *Embase* Excerpta Medica Database; *MEDLINE* Medical Literature Analysis and Retrieval System Online

We identified 144 relevant records, of which 46 unique studies (30 from a previous review [6] and 16 newly identified studies) fulfilled the inclusion criteria (Fig. 2). These 46 studies involved 46,531 patients in 145 different treatment groups which provided mean data for evaluation.

#### Study characteristics

The median study duration was 44 weeks. The majority (87%) of studies did not allow background LABA use, although over half (59%) allowed background ICS treatment. Most had a minimum threshold inclusion criterion of a smoking history of 10 pack-years (80%). The most frequent upper thresholds for the FEV_1_ inclusion criterion were ≤80% (35%) and ≤70% (33%) (Additional file 1: Table S2). The distribution of study treatment groups and patients across treatment categories is provided in Table 1, with most patients receiving LABA (34.5%), LABA + ICS (20.0%) or LAMA (18.5%).

**Table 1:** Distribution of study treatment groups and patients across treatment categories

Treatment categories	Treatment groups		Patients	
	*N*	%	*N*	%
ICS	3	2.07	626	1.35
LABA	51	35.17	16,059	34.51
LABA + ICS	27	18.62	9282	19.95
LABA + LAMA	17	11.72	5842	12.56
LAMA	20	13.79	8618	18.52
Other	3	2.07	299	0.64
Placebo	24	16.55	5805	12.48
Total	145	100	46,531	100

*Abbreviations*: *ICS* inhaled corticosteroid, *LABA* long-acting β_2_-agonist, *LAMA* long-acting muscarinic antagonist

#### Population baseline characteristics

A summary of patient characteristics across all 145 treatment groups from the 46 studies are presented in Table 2. The mean (standard deviation) age of patients was 63.5 (17.4) years and there were more males (68%) than females. The mean percentage predicted FEV_1_ was 45.6% (range: 32.8–60.1%). The baseline characteristic showing the most variability across treatment groups was disease severity as assessed using GOLD grades [2]. The percentage of patients with moderate COPD (GOLD grade 2) ranged from 0 to 99.7% and the percentage of patients with severe or very severe COPD (GOLD grades 3 or 4) ranged from 0 to 100% (Table 2).

**Table 2:** Key baseline characteristics summarized across all study treatment groups (weighted^a^)

Characteristics	Patients evaluated	Population estimate		
	*n* (missing)	Mean (SD)	Median	Min–Max
*N* ^b^	145 (0)	464 (6632)	403	6–1721
Age, years	138 (7)	63.5 (17.4)	63.6	58.8–68.1
Male, %	143 (2)	68.3 (153)	68	43–100
Mean duration of COPD, years	46 (99)	8.0 (37.9)	7.1	5.8–11.3
Current smokers, %	118 (27)	43.2 (127)	42.9	0–65.0
Mean of pack-years of cigarettes	71 (74)	45.2 (98.6)	44.0	34.8–58.5
Mean baseline trough FEV_1_ (L)	84 (61)	1.21 (3.65)	1.22	0.89–1.74
Mean % predicted FEV_1_	97 (48)	45.6 (129)	45.2	32.8–60.1
% GOLD grade 2	78 (67)	29 (540)	19	0–99.7
% GOLD grades 3 and 4	76 (69)	60 (640)	54	0–100
Mean baseline SGRQ score	70 (75)	49.4 (79.4)	48.2	38.4–58.6
Mean BDI score	38 (107)	6.3 (8.4)	6.4	5.1–7.0

*Abbreviations*: *BDI* Baseline dyspnoea index, *COPD* chronic obstructive pulmonary disease, *FEV*_*1*_ forced expiratory volume in one second, *GOLD* Global initiative for chronic Obstructive Lung Disease, SGRQ, St George’s Respiratory Questionnaire, *SD* standard deviation

^a^The statistics calculated take into account the treatment group sample size by applying weights to each of the characteristics described; the weight for each treatment group is proportional to its sample size

^b^The total number of patients randomized in all studies was 46,531

### Correlation analyses between rescue medication use and COPD outcomes

The Pearson correlation coefficients between mean change from baseline in rescue medication use and mean change from baseline in COPD outcomes at 6 months and study end are presented in Table 3.

**Table 3:** Pearson correlation coefficients between rescue medication use and other COPD outcomes

Outcome	Mean change from baseline in number of puffs/day		Mean change from baseline in % of rescue-free days	
	*N*	Pearson correlation coefficient [95% CI], *p*-value	*N*	Pearson correlation coefficient [95% CI], *p*-value
Mean change from baseline in trough FEV_*1*_				
6 months	64	−0.66 [−0.78, −0.49], <0.0001	33	0.43 [0.10, 0.67], 0.0118
Study end	94	−0.74 [−0.82, −0.64], <0.0001	46	0.63 [0.41, 0.77], <0.0001
Mean change from baseline in SGRQ score				
6 months	50	0.60 [0.33, 0.72], <0.0001	18	−0.42 [−0.74, 0.05], 0.077
Study end	75	0.78 [0.67, 0.86], <0.0001	31	−0.70 [−0.84, −0.46], <0.0001
Mean TDI				
6 months	57	−0.43 [−0.62, −0.19], 0.0007	24	0.69 [0.40, 0.86], <0.0001
Study end	54	−0.43 [−0.63, −0.19], 0.0009	24	0.69 [0.40, 0.86], <0.0001
Mean annualized rate of moderate or severe exacerbations				
6 months	17	0.66 [0.27, 0.87], 0.0028	11	Insufficient data
Study end	38	0.66 [0.44, 0.81], <0.0001	24	−0.39 [−0.68, 0.02], 0.0619

These analyses were weighted by study treatment group sample size, and included all trial arms

*Abbreviations*: *CI* confidence interval, *COPD* chronic obstructive pulmonary disease, *FEV*_*1*_ forced expiratory volume in one second, *SGRQ* St George’s Respiratory Questionnaire, *TDI* Transition Dyspnoea Index

**Fig. 3:**
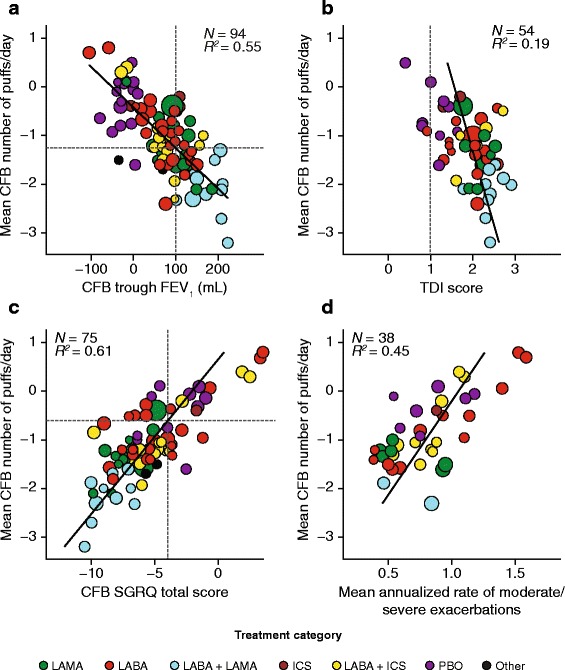
Weighted linear regression bubble plots for associations (study end) between rescue puffs/day and **a** CFB trough FEV_1_; **b** TDI score; **c** CFB SGRQ total score; **d** mean annualized rate of exacerbations. *Abbreviations*: *CFB* change from baseline; *FEV*_1_ forced expiratory volume in one second; *ICS* inhaled corticosteroid; *LABA* long-acting β2-agonist; *LAMA* long-acting muscarinic antagonist; *PBO* placebo; *SGRQ* St George’s Respiratory Questionnaire; *TDI* Transition Dyspnoea Index

The mean change from baseline in number of puffs/day was negatively correlated with mean change from baseline in trough FEV_1_ and TDI at both the 6-month time point (trough FEV_1_: *R* ^2^ = 0.43; TDI: *R* ^2^ = 0.19) and study end (trough FEV_1_: *R* ^2^ = 0.55; TDI: *R* ^2^ = 0.19; Table 3; Fig. 3a and b). To ensure that the presence of two treatment groups considerably larger than the others (from the INVIGORATE study [10]) did not skew the data, the regression data for association between mean change from baseline in number of puffs/day and mean change from baseline in TDI at study end were reanalyzed without these two treatment groups; no significant change in the *R* ^2^ value was observed. A positive correlation was observed between mean change from baseline in puffs of rescue medication per day and both mean change from baseline in SGRQ score and mean annualized rate of moderate or severe exacerbations, at both 6 months (SGRQ score: *R* ^2^ = 0.31; exacerbation rate: *R* ^2^ = 0.44) and study end (SGRQ score: *R* ^2^ = 0.61; exacerbation rate: *R* ^2^ = 0.45; Table 3; Fig. 3c and d).

The overall picture was very similar when rescue medication use was expressed as change in percentage of rescue medication-free days: positive correlations were observed with changes in trough FEV_1_ and TDI score at 6 months (trough FEV_1_: *R* ^2^ = 0.18; TDI: *R* ^2^ = 0.48) and study end (trough FEV_1_: *R* ^2^ = 0.36; TDI: *R* ^2^ = 0.48; Table 3; Additional file 2: Figures S1A and b). A negative correlation was observed with change in SGRQ score at study end (*R* ^2^ = 0.49, Table 3; Additional file 2: Figure S1C); the correlation at 6 months was not significant (*R* ^2^ = 0.18). The correlation with mean annualized rate of moderate or severe exacerbations was moderate but non-significant (*R* ^2^ = 0.15) at study end, while there were insufficient data to assess the correlation at the 6-month time point (Table 3; Additional file 2: Figure S1D).

The Pearson correlation coefficients between rescue medication use and COPD outcomes at baseline are shown in Additional file 1: Table S3. Correlations between mean trough FEV_1_ and both rescue medication use outcomes were significant and large. The correlation between mean number of puffs/day and mean SGRQ score at baseline was significant and moderate. In contrast, the correlations between percentage of rescue medication-free days and mean SGRQ score, and between number of puffs/day and mean baseline dyspnoea index (BDI) were not significant, and there were insufficient data to determine the correlation between percentage of rescue medication-free days and BDI (Additional file 1: Table S3).

### Estimation of rescue medication values corresponding to MCID for COPD outcomes

The regression slope between mean change from baseline in number of puffs/day and mean change from baseline in trough FEV_1_ at study end showed that a change of 100 mL in trough FEV_1_ (the proposed MCID value for the FEV_1_ [11]) was associated with a mean reduction of 1.3 puffs/day (Fig. 3a). Similarly, with the SGRQ, the established MCID of −4 points [12] was associated with a mean reduction of 0.6 puffs/day (Fig. 3c). The corresponding analysis was not possible with the TDI because the established MCID of +1 point [13] fell outside the limits of the regression slope (Fig. 3b). The same pattern was also observed at the 6-month time point (data not shown).

The equation for the regression slope between mean change from baseline in number of puffs/day and annualized rate of moderate or severe exacerbations (annualized exacerbation rate = 1.054 + 0.26 × [rescue medication use in puffs/day]) suggests that a reduction in rescue medication use of -1.0 puffs/day corresponds to a decrease in annualized exacerbation rate of 0.26 events/patient-year.

## Discussion

This analysis showed that rescue medication use, expressed either as change in number of puffs/day or change in the number of rescue medication-free days, is associated with other clinically important outcomes in COPD trials including functional assessments (trough FEV_1_), patient-centered outcomes assessing health status (SGRQ score), dyspnoea (TDI) and annualized rate of exacerbations. This suggests that rescue medication use would be a valid measure to include in health technology assessments.

The primary analysis showed that an increase in trough FEV_1_ from baseline was associated with a decrease in the number of rescue medication puffs/day. The correlation between mean change from baseline in puffs/day and change in trough FEV_1_ at study end (*R* ^2^ = 0.55) was similar to that reported in an analysis of pooled data from three studies in which the correlation (*R* ^2^) at cohort level data was 0.77 [9]. The association between reduction in puffs/day and change in mean SGRQ score was slightly stronger than that seen for FEV_1_ with a positive correlation at study end. The reduction in puffs/day was associated with an improvement in breathlessness as measured by the mean change in TDI focal score at 6 months and study end. However, a stronger association between change in the percentage of rescue-free days and TDI focal score was observed at 6 months and study end. This implies that an increase in rescue medication-free days may be the stronger indicator of a reduction in breathlessness. Reduction in puffs/day was associated with a reduction in the annualized rate of moderate or severe exacerbations at 6 months and study end. Most correlations were the same or slightly stronger from 6 months to study end. This finding could suggest that any data collected in a 6-month study is likely to capture the full efficacy benefit of treatment using most common outcome measures.

Recently, it has been reported that levels of daily short-acting β_2_-agonist use and temporal changes in their use in patients with COPD are likely to be an important risk factor for moderate/severe exacerbations in higher-risk patients [3]. In support of this, our data also highlighted a good correlation between increases or decreases in rescue medication use and annualized moderate or severe exacerbation rate. It was also observed that a change from baseline in rescue medication use of 1.0 puffs/day corresponded to a difference in annualized moderate or severe exacerbation rate of 0.26 events/patient-year. Although a clinically relevant change in annualized rate of moderate or severe exacerbations has yet to be established, recent large comparator exacerbation studies have reported treatment differences between 0.11 and 0.30 events/patient-year in populations with exacerbation rates of at least one event/patient-year at baseline [14–16].

Having established moderate to strong correlations between rescue medication use and other outcomes, we performed a mapping exercise to establish the change in rescue medication use that was associated with established MCIDs in COPD outcome measures. This was not possible with the TDI because the mean improvement in score exceeded the MCID even in patients receiving placebo, which may have been a clinical trial effect. It was possible to obtain an estimate of the reduction in puffs/day associated with the MCID in SGRQ and trough FEV_1_, but there was a two-fold difference between the estimates (−0.6 and −1.3 puffs/day, respectively). Variations in estimates such as these are typically seen with mapping exercises when used to estimate the MCID for a new outcome using different comparators; to address this, ‘triangulation’ is commonly performed (using multiple methods and calculating an average). In this context, it was not possible to obtain an estimate using the TDI, so a possible MCID value for rescue puffs/day remains uncertain.

For all outcomes except mean TDI, correlations with mean changes in number of puffs per day were stronger than correlations with mean changes in the percentage of rescue-free days; indeed, correlations of changes in SGRQ score at 6 months and mean annualized rate of moderate or severe exacerbations at 12 months with changes in the percentage of rescue-free days were not statistically significant. The reason for this observation is not clear. This may reflect the fact that, as independent variables, mean change in lung function, SGRQ total score and overall moderate/severe exacerbation rate have a capacity for small incremental changes in proportion to the mean change in rescue medication puffs/day as a dependent variable. However, for rescue-free days as a dependent variable, movement in a population is more restricted, with many patients grouped at extreme values with 100 or 0% of days rescue-free. As this measure does not reflect the extent of use on days when rescue medication is taken, it might be suggested that it provides a less accurate reflection of disease severity than the mean number of puffs/day. It is also possible that patients may use rescue medication prophylactically (for example, before exercise with the aim of preventing exercise-induced dyspnea); this may skew data on the percentage of rescue-free days to a greater extent than the mean number of puffs/day. The reduced ability of the percentage of rescue-free days to show incremental improvement or deterioration from baseline may more closely align with changes in TDI, with all data points showing relatively restricted mean improvement by 1 to 3 units in virtually all study arms and no mean data points showing deterioration.

This analysis used a large data set obtained in 145 treatment groups containing 46,531 patients from 46 studies, which is a strength. However, a key limitation is that the data were analyzed using meta-techniques, rather than pooled individual patient data. Future research examining patient level data, such as a pooled analysis, is required to gain a better understanding of how levels of rescue medication use and changes from baseline in its use reflect patient burden of disease, using conventional patient-reported outcomes and exacerbation incidence to better estimate a likely MCID for rescue medication use. More research is also needed into whether there are identifiable patient demographics or disease-related factors that can account for differences between individuals in rescue medication use.

As with all systematic reviews, a limitation is the availability of data reported from the primary trials. This is well illustrated by the difference of almost two-fold in the reporting of FEV_1_ data compared with exacerbations. Another limitation of the method used to estimate an MCID for rescue medication use came from the performance of the TDI, for which large improvements were observed even in patients who received placebo, which presumably reflects the operation of a large clinical trial effect on this outcome. One notable omission from this analysis is any correlation with specific COPD symptoms as measured by an instrument such as the Exacerbations of Chronic Pulmonary Disease Tool (EXACT)-Respiratory Symptom (E-RS) measure [17], since this is a newly developed instrument.

## Conclusions

This analysis shows that a reduction in rescue medication use is associated with improvement in trough FEV_1_, breathlessness and health status, and with the rate of exacerbations during the study period. These associations were moderate to strong at a study group level, showing that this outcome may be a surrogate marker of symptomatic benefit in COPD trials. However, confirmation of this conclusion requires individual patient data analysis.

## Additional files

Additional file 1: Table S1. Search strategy for Embase® and MEDLINE® using embase.com platform. **Table S2.** Key study characteristics. **Table S3.** Pearson correlation coefficients between rescue medication use and other COPD outcomes at baseline. (DOC 59 kb)

Additional file 2: Figure S1. Weighted linear regression bubble plots for associations (study end) with percentage of rescue-free days. CFB, change from baseline; FEV_1_, forced expiratory volume in one second; ICS, inhaled corticosteroid; LABA, long-acting β_2_-agonist; LAMA, long-acting muscarinic antagonist; PBO, placebo; SGRQ, St George’s Respiratory Questionnaire; TDI, Transition Dyspnoea Index. (PDF 1370 kb)

## Abbreviations

BDI: Baseline dyspnoea index
CFB: Change from baseline
CI: Confidence interval
COPD: Chronic obstructive pulmonary disease
EMA: European Medicines Agency
Embase: Excerpta Medica Database
FDA: Food and Drug Administration
FEV_1_: Forced expiratory volume in one second
GOLD: Global initiative for chronic Obstructive Lung Disease
HTA: Health Technology Assessment
ICS: Inhaled corticosteroid
LABA: Long-acting β_2_-agonist
LAMA: Long-acting muscarinic antagonist
MCID: Minimal clinically important difference
MEDLINE: Medical Literature Analysis and Retrieval System Online
NIHR: National Institute for Health Research
PBO: Placebo
RCT: Randomized controlled trial
SD: Standard deviation
SGRQ: St George’s Respiratory Questionnaire
TDI: Transition Dyspnoea Index
WHO: World Health Organization
