# Supplementary material for: Rescue medication use as a patient-reported outcome in COPD: a systematic review and regression analysis
Source: Respir Res. 2017 May 8;18:86. doi: 10.1186/s12931-017-0566-1 (PMC5422957; doi:10.1186/s12931-017-0566-1)
Supplement: Supplementary file 1 — Search strategy for Embase® and MEDLINE® using embase.com platform. Table S2. Key study characteristics. Table S3. Pearson correlation coefficients between rescue medication use and other COPD outcomes at baseline. (DOC 59 kb) [file 12931_2017_566_MOESM1_ESM.doc]

# Additional File 1

**Table S1** Search strategy for Embase® and MEDLINE® using embase.com platform

| **No.** | **Query** | **Results** |
| --- | --- | --- |
| #1 | formoterol:ab,ti OR eformoterol:ab,ti OR foradil:ab,ti OR oxis:ab,ti OR 'atimos modulite':ab,ti OR atock:ab,ti OR perforomist:ab,ti OR salmeterol:ab,ti OR serevent:ab,ti OR tiotropium:ab,ti OR spiriva:ab,ti OR 'ba 679 br':ab,ti OR indacaterol:ab,ti OR onbrez:ab,ti OR arcapta:ab,ti OR 'nva 237':ab,ti OR nva237:ab,ti OR (nva NEAR/1 237):ab,ti OR glycopyrronium:ab,ti OR glycopyrrolate:ab,ti OR seebri:ab,ti OR 'enurev breezhaler':ab,ti OR 'aclidinium':ab,ti OR 'tudorza pressair':ab,ti OR 'eklira genuair':ab,ti OR symbicort:ab,ti OR advair:ab,ti OR seretide:ab,ti OR olodaterol:ab,ti OR striverdi:ab,ti OR umeclidinium:ab,ti OR gsk573719:ab,ti OR vilanterol:ab,ti OR gw642444:ab,ti OR qva149:ab,ti OR relovair:ab,ti OR zephyr:ab,ti OR 'anoro ellipta':ab,ti | 7568 |
| #2 | 'chronic obstructive lung disease'/syn | 91847 |
| #3 | copd:ab,ti OR 'chronic obstructive pulmonary disease':ab,ti OR coad:ab,ti OR 'chronic obstructive airway disease':ab,ti OR 'chronic obstructive lung disease':ab,ti OR 'chronic bronchitis':ab,ti OR emphysema:ab,ti | 88147 |
| #4 | #2 OR #3 | 118999 |
| #5 | #1 AND #4 | 2736 |
| #6 | random*:ab,ti OR placebo*:ab,ti OR single NEAR/1 blind* OR double NEAR/1 blind* OR triple NEAR/1 blind* | 1094372 |
| #7 | 'retracted article'/exp | 6868 |
| #8 | #6 OR #7 | 1101083 |
| #9 | #5 AND #8 | 1274 |
| #10 | 'animal'/de OR 'nonhuman'/de OR 'animal experiment'/de AND 'human'/de | 1327627 |
| #11 | 'animal'/de OR 'nonhuman'/de OR 'animal experiment'/de | 6419973 |
| #12 | #11 NOT #10 | 5092346 |
| #13 | #9 NOT #12 | 1274 |
| #14 | #9 NOT #12 AND ([conference review]/lim OR [editorial]/lim OR [letter]/lim OR [note]/lim OR [review]/lim) | 165 |
| #15 | #13 NOT #14 | 1109 |
| #16 | #13 NOT #14 AND ([english]/lim | 1065 |
| #17 | #13 NOT #14 AND ([english]/lim AND [1-4-2014]/sd | 187 |

**Table S2 Key study characteristics**

| **Characteristics** | **All studies (N=46)** |
| --- | --- |
| Trial duration |  |
| Median, weeks | 44 |
| Background LABA treatment allowed, n (%) |  |
| No | 40 (87.0) |
| Yes | 0 (0) |
| Missing | 6 (13.0) |
| Background ICS treatment allowed, n (%) |  |
| No | 17 (37.0) |
| Yes | 27 (58.7) |
| Missing | 2 (4.3) |
| Lower threshold of pack-years of cigarettes, n (%) |  |
| ≥10 | 37 (80.4) |
| ≥15 | 2 (4.3) |
| ≥20 | 6 (13.0) |
| Missing | 1 (2.2) |
| Upper threshold of percentage predicted FEV_1_ |  |
| ≤50% | 8 (17.4) |
| ≤60% | 0 (0.0) |
| ≤65% | 4 (8.7) |
| ≤70% | 15 (32.6) |
| ≤80% | 16 (34.8) |
| ≤85% | 1 (2.2) |
| Missing | 2 (4.3) |

FEV_1_, forced expiratory volume in one second; ICS, inhaled corticosteroid; LABA, long-acting β_2_‑agonist; LAMA, long-acting muscarinic antagonist.

Percentage values may not sum to 100.0 exactly due to rounding.

**Table S3** Pearson correlation coefficients between rescue medication use and other COPD outcomes at baseline

| **Outcome** | **Mean number of puffs/day** | | **Mean % of rescue-free days** | |
| --- | --- | --- | --- | --- |
|  | **N** | **Mean Pearson correlation coefficient**  **[95% CI], p-value** | **N** | **Mean Pearson correlation coefficient**  **[95% CI], p-value** |
| **Mean trough FEV_1_ (mL)** | 64 | -0.59 [-0.73, -0.41],<0.0001 | 34 | 0.63 [0.38, 0.80], <0.0001 |
| **Mean SGRQ score** | 50 | 0.46 [0.20, 0.65], 0.0008 | 16 | -0.48 [-0.79, 0.02], 0.0572 |
| **Mean BDI** | 28 | 0.32 [-0.05, 0.62], 0.0924 | 13 | Insufficient data |

BDI, Baseline Dyspnoea Index; CI, confidence interval; COPD, chronic obstructive pulmonary disease; FEV_1_, forced expiratory volume in one second; SGRQ, St George’s Respiratory Questionnaire.

These analyses were weighted by study treatment group sample size.
