# Supplementary material for: Rescue medication use as a patient-reported outcome in COPD: a systematic review and regression analysis
Source: Respir Res. 2017 May 8;18:86. doi: 10.1186/s12931-017-0566-1 (PMC5422957; doi:10.1186/s12931-017-0566-1)
Supplement: Supplementary file 2 — Weighted linear regression bubble plots for associations (study end) with percentage of rescue-free days. CFB, change from baseline; FEV1, forced expiratory volume in one second; ICS, inhaled corticosteroid; LABA, long-acting β2-agonist; LAMA, long-acting muscarinic antagonist; PBO, placebo; SGRQ, St George’s Respiratory Questionnaire; TDI, Transition Dyspnoea Index. (PDF 1370 kb) [file 12931_2017_566_MOESM2_ESM.pdf]

Figure S1: Weighted linear regression bubble plots for associations (study end) between percentage of rescue-free days and: A) CFB trough FEV_1; B) TDI score; C) CFB SGRQ total score; D) mean annualized rate of exacerbations

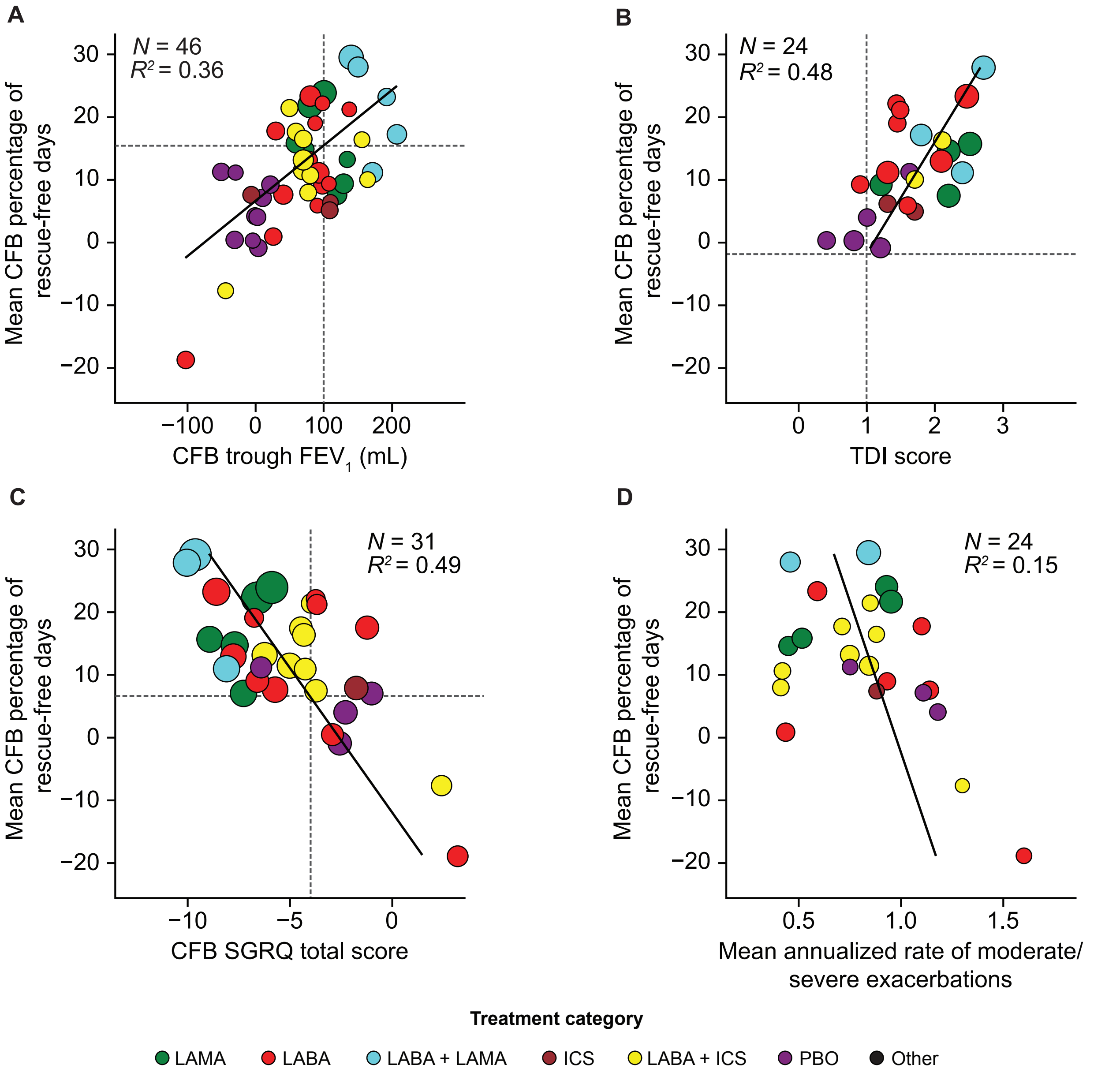

CFB, change from baseline; FEV_1, forced expiratory volume in one second; ICS, inhaled corticosteroid; LABA, long-acting β_2-agonist; LAMA, long-acting muscarinic antagonist; PBO, placebo; SGRQ, St George's Respiratory Questionnaire; TDI, Transition Dyspnoea Index.
